# Effect of antiretroviral therapy on bone turnover and bone mineral density in men with primary HIV-1 infection

**DOI:** 10.1371/journal.pone.0193679

**Published:** 2018-03-09

**Authors:** Mariska C. Vlot, Marlous L. Grijsen, Jan M. Prins, Renate T. de Jongh, Robert de Jonge, Martin den Heijer, Annemieke C. Heijboer

**Affiliations:** 1 Department of Clinical Chemistry, Endocrine Laboratory, VU University Medical Center, Amsterdam, the Netherlands; 2 Department of Internal Medicine, Endocrinology, VU University Medical Center, Amsterdam, the Netherlands; 3 Department of Internal Medicine, Infectious Diseases, Center for Infection and Immunity, Academic Medical Center, Amsterdam, the Netherlands; 4 Department of Dermatology, Leiden University Medical Center, Leiden, the Netherlands; 5 Department of Clinical Chemistry, Endocrine laboratory, Academic Medical Center, Amsterdam, the Netherlands; Azienda Ospedaliera Universitaria di Perugia, ITALY

## Abstract

**Introduction:**

Previous studies indicate that human immunodeficiency virus (HIV)-infection and combination antiretroviral therapy (cART) can affect bone turnover. Furthermore, HIV-infected patients have lower bone mineral density (BMD) compared to a healthy reference population.

**Objective:**

To evaluate the longitudinal effect of HIV-infection and cART on bone turnover markers (BTMs) and BMD in men with primary HIV-infection (PHI).

**Design, methods:**

Thirty-five PHI-men were divided into two groups, those that received cART for the first time (n = 26) versus no-cART (n = 9). Dual-energy X-ray absorptiometry (DXA) was performed on femoral neck (FN), total hip (TH) and lumbar spine (LS) and BTMs (P1NP, alkaline phosphatase, osteocalcin, ICTP and CTX) were measured at baseline and follow-up.

**Results:**

At baseline, the median CD4+ T-cell count was 455 cells/mm3 (IQR 320–620) and plasma viral load 5.4 log10 copies/mL (IQR 4.7–6.0) in the cART treated group, compared to 630 (IQR 590–910) and 4.8 (IQR 4.2–5.1) in the untreated group. The median follow-up time was 60.7 weeks (IQR 24.7–96.0). All BTMs, except ICTP, showed a significant increase during cART versus no changes of BTMs in the untreated group. FN and TH BMD showed a significant decrease in both groups. LS BMD did not change in both groups.

**Conclusion:**

Bone turnover increased in PHI-men treated with cART which was accompanied by a decrease in FN and TH BMD. No increase of bone turnover was seen in untreated PHI-men. Our study suggests that cART results in increased bone turnover and decreased BMD of the hip in PHI-men.

## Introduction

Studies have shown that HIV-infected patients often have a decreased bone mineral density (BMD) compared to NHANES reference groups, with an estimated prevalence of around 67% [1–4]. The accompanying higher risk of osteopenia, osteoporosis and fractures implies that monitoring their BMD is highly important [5–9]. Bone loss in HIV-infected patients seems to result from a combination of several contributing factors such as a lower vitamin D status, lower BMI and higher usage of alcohol and tobacco [7,10–12]. Also, combination antiretroviral therapy (cART) is associated with an even stronger decrease in BMD in HIV-infected patients [3,4,10,13]. A decline of 2–4% of the total bone mass upon starting cART is known to occur within the first 2 years of cART [14,15]. Furthermore, a recent review showed that patients on cART were up to 2.5 times more prone to have T-scores <1 compared to untreated HIV-patients [11].

BMD measurements are assessed by a dual energy X-ray absorptiometry (DXA) scan. However, this method allows to estimate changes in bone mineral content which occur over years. In contrast, bone turnover markers (BTMs) reflect dynamic and short-term changes in bone remodeling. Therefore, the actual bone turnover is best reflected by measurements of BTMs as bone remodelling is a continuous and variable process. Bone resorption is displayed by resorption markers such as c-telopeptide crosslink of type 1 collagen (CTX) that is produced by osteoclasts and bone formation is reflected by formation markers such as procollagen type 1 N-terminal propeptide (P1NP) that is produced by osteoblasts. With regard to bone turnover, inflammatory cytokines and possibly viral HIV-proteins are thought to increase the activity of osteoclasts resulting in increased bone resorption [2,16,17]. Simultaneously, osteoblast activity is negatively influenced by HIV-proteins [18] and treatment with cART may result in increased bone turnover [4,9,14,19–21]. Altogether, these factors contribute to a lower BMD in HIV-infected persons.

A previous study in therapy-naïve primary HIV-infected (PHI) men, showed that bone turnover did not differ between those with a normal or a reduced BMD [10]. However, most other studies show higher BTMs in PHI-men [3,4,6,10,22–24]. These heterogeneous data might be caused by the choice of BTM, pre-analytical differences (for instance time of the blood withdrawal, fasting sample or not), the applicable reference interval of the BTM, or differences in patient characteristics (for instance males versus females). To date, the course and duration of the increase of BTMs during cART is not fully elucidated [20,25]. Therefore, the aim of this study is to assess the course of bone turnover and BMD in treated and untreated PHI-men.

## Methods

### Subjects and treatment protocol

Patients were selected from the PRIMO-SHM trial, a prospective multicenter cohort study in PHI-men from the Amsterdam Medical Center (AMC) in Amsterdam, the Netherlands, between February 2008 and October 2009, trial number ISRCTN59497461 [10,26]. The main inclusion criteria of this trial were age over 18 years and laboratory evidence of PHI, defined as having detectable plasma HIV-1 RNA with a negative or indeterminate Western blot, or in case of a positive Western blot, a documented negative HIV-RNA test within the previous180 days [10,26]. The study was approved by the Ethical Committee of the AMC. All patients provided written informed consent.

For the current study, patients were selected if they had (i) measurement of the BTMs P1NP, alkaline phosphatase (ALP), osteocalcin (OC), and/or cross-linked carboxyterminal telopeptide of type I collagen (ICTP), CTX, at baseline and/or follow-up, and (ii) DXA-scan performed at baseline and/or follow-up. Patients were excluded in case they (A) suffered from medical conditions that possibly affected bone metabolism such as hypercalcaemia or recent corticosteroid therapy for at least three months, (B) reported earlier use of cART before entering the study or (C) if they needed to (re)start cART during the study because of decreasing CD4+ T-cell counts. Of the total of 43 patients, 35 eligible patients were included in this study.

The patients were divided into two groups, those who had initiated cART during PHI and those who remained untreated. Patients were treated with cART in case of confirmed CD4+ T-cell count < 350 cells/mm^3^ or symptomatic HIV-disease. Twenty-six patients received early cART and nine patients remained untreated. Patients whom received cART were treated according to the PRIMO-SHM trial protocol [26]. Briefly, patients received a combination of emtricitabine (FTC), a nucleoside-analogue reverse transcriptase inhibitor (NRTI), tenofovirdisoproxil (TDF), a nucleotide-analogue transcriptase inhibitor, efavirenz (EFV) a non-nucleoside reverse-transcriptase inhibitor, and a combination of lopinavir and ritonavir, both protease inhibitors (PI). Lopinavir and ritonavir were discontinued when plasma viral load reached <50 log_10_ copies/mL. In case of drug resistance or side effects an adjusted cART combination was prescribed.

### Measurements

#### General

Body weight and height were measured when performing the DXA-scan. Viral load and CD4+ T-cell count were measured at baseline. Fractures, calcium or vitamin D supplementation, smoking and alcohol use were also evaluated at baseline.

#### Bone turnover markers

The formation markers P1NP, ALP and OC and resorption ICTP and CTX and were measured in serum after an overnight fast. P1NP and ICTP were measured using a radioimmunoassay (RIA) (both from Orion Diagnostica, Espoo, Finland) with an intra-assay coefficient of variation (CV) of 4–8% and 4–6%, respectively, and a lower limit of quantitation (LOQ) of 5 μgram/L and 1 μgram/L, respectively. CTX was measured using an electro-chemiluminescence immunoassay (ECLIA) (Roche diagnostics, Almere, the Netherlands) with a CV of <8% and LOQ of 10 ng/L. OC was measured using an immunometric assay (Biosource, Nivelles, Belgium) with an intra-assay CV of <5% and LOQ of 0.4 nmol/L. ALP was measured using a spectrophotometric assay (Roche Diagnostics, Almere, the Netherlands) with an interassay CV of 3% and LOQ of 5 IU/L.

#### Bone densitometry (DXA-scan)

A DXA-scan (Hologic QDR 4500W, Hologic Inc, Bedford, MA, USA) was used to measure BMD of the lumbar spine (L1-L4) (LS), femoral neck (FN) and total hip (TH) of the non-dominant hip. Follow-up data on DXA-scans were available until December 2011. To evaluate osteopenia or osteoporosis status, T-scores and Z-scores were calculated based on the NHANES reference database. Based on WHO criteria a T-score between minus 1 and 2.5 SD reflects osteopenia and minus 2.5 SD or less reflects osteoporosis. As this study comprehends generally younger patients who are supposed to have reached BMD values around the peak bone mass the Z-score is also displayed. The Z-score reflects the BMD SD of the patient compared to healthy age-matched controls with a Z-score of minus 2 SD or less reflecting osteoporosis.

#### Statistics

Stata/SE 14.0 software (StataCorp, LP) was used for statistical analysis. Normality was tested by normality plots. Wilcoxon, Mann-Whitney U, Kruskall-Wallis or T-tests were used based on whether parametric or nonparametric tests were applicable. The median and interquartile range (IQR) were described, unless otherwise specified. Correlations were performed using a Spearman model. Furthermore, a regression model based on linear regression was used. Changes in absolute values between baseline and follow-up of BMD and BTM values were calculated as deltas (Δ) with corresponding 95% confidence intervals (CI) and *p*-values. A *p*-value of ≤0.05 was regarded as significant.

## Results

### Study population

The baseline characteristics of the 35 included patients are displayed in Table 1 and **S1 File**. The CD4+ T-cell count at baseline was lower in the treated versus the untreated group (95% CI 38–433, *p* = 0.02). To adjust for the difference in CD4+ levels between the two groups separate linear regression analyses were performed containing the outcome measures BMD, T- and Z-scores and all bone turnover markers. These analyses however did not affect the results (all *p-*values > 0.05, therefore data not shown). Only one patient in the treated group used vitamin D supplementation. All treated patients received a regimen containing TDF; 76.9% (n = 20) received the triple-class drug regimen according to the study protocol. The total median follow-up time of our study was 60.7 weeks (IQR 24.7 – 96.0), with a longer follow-up time of the untreated group (median 96.6 weeks (IQR 37.7 – 97.4)) compared to the treated group. No hip or vertebral fractures were reported in both groups and no bisphosphonates were used. In the treated group an increase in median BMI was seen from 22.4 (IQR 21.3 – 25.6) to 23.4 (IQR 21.9 – 26.5) (p = 0.02), during follow-up. The untreated group showed a slight but not significant increase of BMI during follow-up.

**Table 1:** Baseline results of PHI-men.

Total n = 35	cART treated, n = 26	Untreated, n = 9	p-value
***General***			
Age (y) mean/SD	39.0 (9.9)	39.5 (9.1)	0.97
BMI	22.4 (21.3–25.6)	23.4 (21.2–24.0)	0.94
Ethnicity (%) Caucasian	84.6	100	0.22
MSM (%)	88.5	100	0.29
Current smoking (%)	38.5	44.4	0.76
Excessive alcohol use* (%)* defined as using ≥ 3 U/day	11.5	0	0.24
Vitamin D deficiency (≤50 nmol/L)(%) Vitamin D supplementation use (%)	30.8 3.9	44.4 0	0.46 0.56
***HIV parameters***			
CD4+ T-cell count (cells/mm^3^)	455 (320–620)	630 (590–910)	0.02*
Plasma HIV-RNA (log_10_ copies/mL)	5.4 (4.7–6.0)	4.8 (4.2–5.1)	0.40
Duration of cART therapy (weeks)	60.3 (24.4–90.9)	N.A.	N.A.
***Bone turnover markers***			
P1NP (μg/L)	37 (28–46)	41 (38–53)	0.27
ALP (U/L)	66 (60–72)	71 (59–88)	0.85
OC (nmol/L)	7.3 (4.3–10.3)	8.8 (2.7–11.2)	0.76
ICTP (μg/L)	3 (2.6–4.2)	3 (2.7–3.1)	0.52
CTX (ng/L)	304.5 (146–384)	191 (124–358)	0.57
***Bone mineral density***			
BMD Femoral neck (g/cm^2^)	0.840 (0.771–0.941)	0.806 (0.736–0.879)	0.48
BMD Total hip (g/cm^2^)	0.968 (0.886–1.068)	1.00 (0.904–1.074)	0.61
BMD Lumbar spine (g/cm^2^)	0.962 (0.872–1.111)	0.995 (0.931–1.023)	0.73
T–score Femoral neck Z-score Femoral neck T–score Total hip Z-score Total hip T–score Lumbar spine Z-score Lumbar spine	− 0.7 (− 1.2 to 0.1) − 0.3 (− 0.9 to 0.3) − 0.4 (− 1.0 to 0.2) − 0.2 (− 0.8 to 0.3) − 1.2 (− 2.0 to 0.2) − 1.1 (− 1.9 to 0.3)	− 1.0 (− 1.5 to 0.4) − 0.4 (− 1.0 to 0.1) − 0.3 (− 0.9 to 0.3) − 0.1 (− 0.5 to 0.5) − 0.9 (− 1.5 to −0.6) − 0.8 (− 1.4 to −0.6)	0.48 0.70 0.64 0.49 0.81 0.73
Osteopenia (%)**in either hip or spine	38.5	44.4	0.63
Osteoporosis (%)**in either hip or spine	15.4	11.1	0.76

Data are expressed as median/IQR, or percentages unless specified otherwise. Abbreviations: PHI = Primary HIV Infection, cART = combination anti-retroviral therapy, N.A. = not applicable, BMI = body mass index, MSM = men who have sex with men, BMD = bone mineral density, P1NP = procollagen type I propeptide, ALP = alkaline phosphatase, OC = osteocalcin, ICTP = cross-linked carboxyterminal telopeptide of type I collagen, CTX = C-terminal telopeptide of type 1 collagen.

* = p ≤ 0.05

### Bone turnover markers

**Fig 1:**
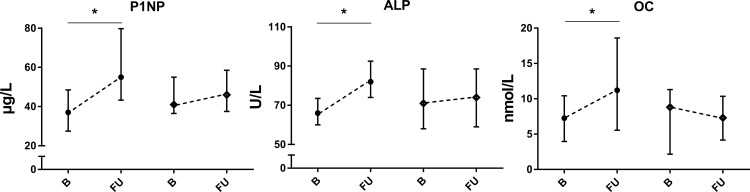
Bone formation markers in PHI-men, * = significant difference p ≤ 0.05 of follow-up versus baseline, ● = cART treated group, ♦ = untreated group, B = baseline, median and IQR, FU = follow-up, median and IQR.

**Fig 2:**
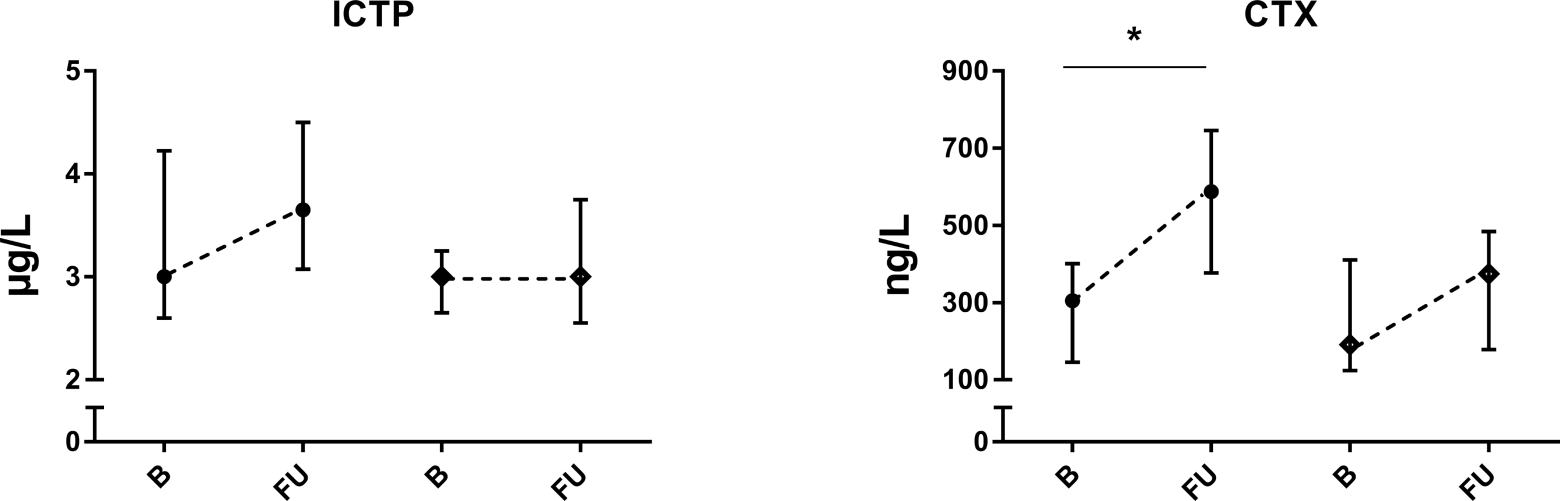
Bone resorption markers in PHI-men, * = significant difference p ≤ 0.05 of follow-up versus baseline, ● = cART treated group, ♦ = untreated group, B = baseline, median and IQR, FU = follow-up, median and IQR.

All BTMs except ICTP increased during cART. P1NP had a mean increase of 22 μg/L (95% CI 9.9–33.3, *p* = 0.0003), ALP of 13.7 U/L (95% CI 5.3–22.1, *p* = 0.0006), OC of 6.9 nmol/L (95% CI 2.2–11.6, *p* = 0.0022), and lastly, CTX showed a mean increase of 225 ng/L (95% CI 113.6–337, *p* = 0.0012). Only the CTX concentration was significantly higher in the treated group compared to the untreated group at follow-up (*p* = 0.02). In the untreated group no changes in the BTMs were found. All changes between baseline and follow-up of BTMs are displayed in Figs 1 and 2.

### BMD measurements

53.9% of the patients in both treated and untreated group had either osteopenia or osteoporosis of the hip or spine at baseline. Osteopenia was more prevalent than osteoporosis in both groups. At baseline, no differences were observed between the different anatomical BMD sites in the treated verses untreated group. During treatment FN and TH BMD decreased with–a0.044 g/cm2 (95% CI–0.067 to–0.020, *p* = 0.002) and–0.042 (95% CI–0.067 to–0.020, *p* = 0.0003). In the untreated group a decrease of FN of–0.019 (95% CI–0.038 to 0.000, *p* = 0.05) and decrease of TH of–0.039 (95% CI–0.063 to–0.014, *p* = 0.03) was seen. LS BMD did not change in both groups. In line with the BMD, also T- and Z-scores changed during follow-up. T-scores of FN and TH decreased with–0.32 (95% CI–0.50 to–0.15, *p* = 0.002) and–0.28 (95% CI–0.38 to–0.18, *p* = 0.0002) in the treated group. The Z-score of FN decreased with–0.28 (95% CI–0.45 to–0.10, *p* = 0.006) and the Z-score of TH decreased with–0.25 (95% CI–0.36 to–0.14, *p* = 0.0003) in the treated group. In the untreated group T-scores decreased as well in the FN with–0.15 (95% CI–0.31 to 0.01, *p* = 0.05) and in the TH with–0.23 (CI 95% - 0.42 to 0.05, *p* = 0.04). Only the Z-score of TH decreased with–0.25 (95% CI–0.41 to–0.09, *p* = 0.03) Again, LS T- and Z-scores did not change in both groups. No significant differences between treated and untreated group were found at follow-up. All measurements at baseline and follow-up of BMD, T- and Z-scores are displayed in Table 2. Only TH Z-score and OC at baseline displayed a negative association (rho - 0.36, *p* = 0.04). No other significant correlations were found between any of the BTMs and BMD measurements or T-scores in the treated and untreated PHI-men.

**Table 2:** Bone mineral density in PHI-men, median and IQR.

	cART treated group		Untreated group	
	*baseline*	*follow-up*	*baseline*	*follow-up*
**Femoral neck**				
BMD (g/cm^2^)	0.840 (0.771–0.941)	0.781 (0.727–0.902)*	0.806 (0.736–0.879)	0.785 (0.688–0.892)*
Z-score	- 0.3 (− 0.9–0.3)	− 0.5 (− 1.0–0.3)*	− 0.4 (− 1.0–0.1)	− 0.4 (− 1.1–0.1)
T-score	− 0.7 (− 1.2–0.1)	− 1.1 (− 1.5 to −0.2)*	− 1.0 (− 1.5 to −0.4)	− 1.1 (− 1.8 to −0.3)*
**Total hip**				
BMD (g/cm^2^)	0.968 (0.886–1.068)	0.936 (0.839–1.017)*	1.0 (0.904–1.074)	1.0 (0.884–1.042)*
Z-score	− 0.2 (− 0.8–0.3)	− 0.5 (− 1.2–0.1)*	− 0.1 (0.5 to −0.5)	− 0.1 (− 0.7–0.2)*
T-score	− 0.4 (− 1.0–0.2)	− 0.6 (− 1.3 to −0.1)*	− 0.3 (− 0.9–0.3)	− 0.3 (− 1.0–0.1)*
**Lumbar spine**				
BMD (g/cm^2^)	0.962 (0.872–1.111)	0.959 (0.876–1.012)	0.995 (0.931–1.023)	0.970 (0.915–1.031)
Z-score	− 1.1 (− 1.9–0.3)	− 1.2 (− 2.0 to −0.2)	− 0.8 (− 1.4 to −0.6)	− 1.1 (− 1.3 to −0.5)
T-score	− 1.2 (− 2.0–0.2)	− 1.2 (− 2.0 to −0.6)	− 0.9 (− 1.5 to −0.6)	− 1.1 (− 1.6 to −0.5)

* = significant difference p ≤ 0.05 of follow-up versus baseline.

## Discussion

Our study explored the effect of HIV-infection and the use of cART on bone turnover and BMD in PHI-men. In men treated with cART, bone turnover increased accompanied by a decrease in BMD of the FN and TH. In the untreated group a slight decrease in FN and TH BMD was observed as well. Nevertheless, bone turnover did not change.

### Bone turnover in PHI-men

P1NP, ALP, OC and CTX levels increased in the treatment group, only ICTP did not change. ICTP is also secreted by other tissues than bone which have masked the effect of cART. Literature reports that the same [7,14,19,21,22,27] and also other BTMs such as bone specific alkaline phosphatase (BALP), pyridoline and deoxypyridinoline crosslinks, may increase during cART [9,24,28]. A decrease of CTX was also described in one study [28], although CTX measurements were performed after several years of cART treatment in this study. Peak levels of BTMs are generally seen with 12–48 weeks after initiation of cART therapy, followed by a plateau phase, which persists for several years during ongoing cART therapy [4,19,20,24,25,27]. In our study, both bone resorption and formation markers increased within the median treatment period of 60 months of cART. The increased bone turnover in the treated group could result from TDF containing cART which is known to mediate mitochondrial dysfunction resulting in cytokine production that stimulate osteoclast maturation and therefore promotes bone resorption [16,18,23]. TDF containing cART is associated with higher levels of CTX, P1NP and OC and BALP compared to cART without TDF [18,20]. Furthermore, it is known that the activity of osteoclasts can increase while osteoblastogenesisis decreases due to PI containing cART, resulting in higher concentrations of pyridoline and deoxypyridinoline crosslinks, which resembles a state of increased bone turnover [28]. On the other hand, PHI itself is thought to limit the function of osteoblasts and stimulate the osteoclasts which may have influenced the bone turnover and BMD as well [17,26]. However, our study questions this direct effect of chronic inflammation due to HIV itself on bone turnover, since we found no changes in BTMs during follow-up in the untreated PHI group.

### Bone mineral density in PHI-men

At baseline, BMD and T-scores were lower in treated and untreated PHI-men compared to the reference group of NHANES, which is in line with a previous study [11]. The lower BMD can result from several risk factors that are known to attribute to lower BMD such as decreased physical activity, drug use, smoking and alcohol consumption, which were described more prevalent in men who have sex with men (MSM) than hetero-sexual men, and dispose high prevalence in our study population as well [11,18,26,29]. Interestingly, Grijsen et al. described a lower BMD in both HIV-positive, but also HIV-negative MSM, indicating that the lower BMD could be pre-existent in MSM and therefore be unrelated to PHI itself [10,30].

During follow-up BMD and T-scores of the hip decreased significantly in both groups. This raises concern as baseline BMD and T-scores were already low. The hip BMD decreased less in the untreated group compared to the treated group. Several underlying mechanisms could result in the further decrease of BMD such as persistent vitamin D deficiency, continued immune activation and cytokine release induced by the HIV-infection itself, and cART effects in the treated group [6,10,12,20,23,26, 28,31]. Currently, the European HIV guidelines indicate to screen only persons who are at risk for vitamin D deficiency and not all PHI-patients [32]. Nevertheless, almost 40% of our total study population had a vitamin D deficiency at baseline. With regard to the effect of HIV-infection itself on bone, high levels of viral load which are often seen in PHI, are known to decrease osteoblast activity and increase osteoclast function, both resulting in decrease of BMD [10,23,33,34]. This mechanism could explain the decreased BMD in both the treated and untreated group of PHI-men. In contrast, other studies argue that inflammation cannot be seen as a key mediator of decreasing BMD in a group of cART receiving HIV-men [7]. Interestingly, the decrease of BMD in the treated group was more pronounced in hip region compared to the LS, where only a slight but not significant decrease of BMD was found. Several other studies reported a similar pattern of decrease in BMD, even after several years of cART therapy [17,19,20,28]. Other studies, however, describe a decrease of both FN and LS BMD [4,8,16,22,30] or did not report a reduction in BMD after start of cART [23,28]. The timing of the DXA-scan might play a role in explaining these differences as a decrease of BMD is seen especially after 24 weeks and up to 96 weeks after start of cART [9,24,35]. The decrease of BMD is generally attenuated between week 48 and 96 [4,8,11,15]. This stabilization or even slight increase of BMD is thought to result mainly from a recovered balance in bone turnover during continuous long-term cART use [27]. Therefore, in our study the maximum effect of cART on the hormonal active trabecular bone of the LS might have not been detected yet as the follow-up DXA-scan was repeated relatively early after start of cART and furthermore the number of included patients might have been too small to be able to detect changes.

Finally, with regard to the impact of cART on BMD, it is known that TDF is associated with a up to 1–3% stronger decrease of BMD compared to other NRTI containing cART, both in already treated and untreated PHI-men [8,11,35,36]. In our study, all patients in the treated group received a TDF containing cART regime. TDF is thought to cause proximal tubulopathy in the kidneys which results in renal phosphate wasting, osteomalacia and is therefore considered as mediator of the detrimental effect on BMD in HIV [13,15,28,36]. This is reinforced by the fact that TDF is also thought to change vitamin D homeostasis by increasing vitamin D binding protein and decreasing 1.25diOHD levels and osteoblast function [15,18,36], both resulting in increased bone turnover and lower BMD as well. Recently, tenofovir alafenamide (TAF) containing cART, which is the successor of TDF, was shown to display less bone toxicity and result in a smaller decrease of BMD in HIV-patients resulting in better long-term bone safety [37,38,39]. Based on these findings we suggest to be cautious starting TDF containing cART in HIV-patients particularly in those patients who already have osteopenia, osteoporosis or high fracture risk due to other causes.

Currently almost all PHI-patients start cART therapy directly in accordance with recent guidelines [32]. Therefore, this study offers an unique overview of the effect of HIV-infection and cART on bone turnover and BMD as our study shows data of both treated but also non-treated PHI-men. Other strengths of this study are the use of a wide panel of both bone resorption and formation markers, all measured in fasting samples with a standardized method, and a homogenous study population of patients who were all included from a highly specialized academic medical center. This study also has several limitations: data is lacking about the physical activity or possible sedentary lifestyle of our patients. In addition, follow-up data of vitamin D supplementation use or smoking was not available. We describe a relatively small group of almost all Caucasian men, which limits the extrapolation of findings to women or older MSM, in which osteopenia, osteoporosis and increased fracture risk might be even more prevalent. Lastly, the study design did not allow testing for possible causality of HIV itself with regard to bone turnover and BMD, as a control group of MSM with equal behavioral risk factors but who were HIV-negative was lacking.

In conclusion, bone turnover increases and FN and TH BMD decreases in PHI-men during treatment with TDF containing cART. These findings reinforce the matter to strongly consider alternatives for TDF containing cART in men with PHI who already have osteopenia or osteoporosis at time of diagnosis of HIV. In addition, this research stresses the need of evaluation of vitamin D levels and consideration of calcium and vitamin D supplementation to ensure the best possible bone health during TDF containing cART in PHI-men. Finally, as the incidence of HIV and need of cART are still increasing, this will result in more patients with osteopenia and osteoporosis and therefore studies regarding the effect of HIV and its treatment on bone turnover, BMD and fracture risk are warranted.

## Supporting information

S1 File (XLS)
